# Sir Guy Calthrop's Appeal to the Medical Profession

**Published:** 1918-09-14

**Authors:** Guy Calthrop


### The Coal Shortage.

### SIR GUY CALTHROP'S APPEAL TO THE MEDICAL PROFESSION.

"THE country is faced with a serious coal shortage, and I appeal to all medical men whose influence is so great to do their utmost to bring the facts to the notice of all with whom they come in contact, with the view to enlisting the services of every man, woman and child in this country in one united effort to save coal.

Coal is the key industry of Great Britain and the Allies, and the outlook to-day is very much more serious than is generally realised. The causes of the shortage are:

1. The call to the Colours of 75,000 miners to meet the peril of the German offensive in March; and 2. The almost complete stoppage of the mines in Northern France as a direct result of the German advance in the West.

Coal is the source of power; it makes gas, electricity, and steam. It drives the ships and it drives the trains. The coal of England must be shared with our Allies—France, Italy, and America. It helps to carry the American Army to France. It helps them to move their Army while in France, and it keeps their soldiers warm. It is sold to neutrals to buy shipping to bring American troops over, and is exchanged for food which would otherwise go to Germany.

Coal is the source of power wanted to end the war. Coal burned in a house in excess of absolute need is power wasted. It is, therefore, the duty of everyone to save coal, because to save coal is to save lives. Except among the poorest houses there will not be a dwelling in Great Britain this winter with as much coal as it would like to burn. Self-denial is called for. England to-day is short of 36,000,000 tons of coal. By the system of household rationing we hope to save 9,000,000 tons of coal.

Twenty-seven million tons, therefore, remain still to be found. This deficit can be *reduced*—not made good—only if the miners get more coal, and if householders use less than their ration. Even then the supplies of coal to industrial works will be short. This will mean that woollen manufacturers, pottery manufacturers, fabric dyers, bleachers and others may have their business seriously curtailed, and their workpeople consequently must suffer.

Notwithstanding economies already made in these directions we are still on the danger line, and the facts cannot be too insistently and too often brought to the notice of the people of this country. The stocks of our munition works are being eaten into, gas and electric companies are crying for coal to build up their stocks against the winter months. These stocks are not being accumulated at the present time; they are being drawn upon. And we have not been able to fulfil our coal obligations to our Allies.

The miners' leaders have promised to do their utmost to induce the men to increase the output, and the public are being asked to do their part in reducing the consumption of coal, coke, gas, and electricity to a minimum. It is a race with winter. The miners and mine managers and owners can help the country to win through. Every consumer should try to manage on three-quarters of his ration. The quarter saved will help to keep our brave soldiers warm."

